# Expression of wild-type p53-induced phosphatase 1 in diabetic epiretinal membranes

**DOI:** 10.18632/oncotarget.16683

**Published:** 2017-03-29

**Authors:** Jiping Xu, Haibin Zhong, Ling Cui, Qianqian Lan, Lifei Chen, Wenjing He, Yu Wu, Li Jiang, Hui Huang, Xin Zhao, Li Li, Siming Zeng, Min Li, Fan Xu

**Affiliations:** ^1^ Department of Ophthalmology, People's Hospital of Guangxi Zhuang Autonomous Region, Nanning, Guangxi, People's Republic of China

**Keywords:** Wip1, epiretinal membranes, proliferative diabetic retinopathy, NF-κB, glial cell, Pathology Section

## Abstract

**Objective:**

The aims of the present study were to investigate the expression and distribution of Wild-type p53-induced phosphatase 1 (Wip1) in diabetic patients with proliferative diabetic retinopathy (PDR) with epiretinal membranes (ERMs) meanwhile analyze the colocalization of Wip1 and nuclear factor kappa-B (NF-κB) p65 in ERMs.

**Methods:**

ERMs samples were collected from patients with PDR (PDR group) or non-diabetic patients with idiopathic epiretinal membranes (iERMs) (control group) during pars plana vitrectomy. Real-Time PCR analysis was carried out to examine the mRNA expression of Wip1 in ERMs. Immunohistochemical analysis and Immunofluorescent analysis were performed to detect the protein expression of Wip1 in ERMs. Double immunofluorescent staining was performed to detect the colocalization of Wip1 and glial fibrillary acidic protein (GFAP) (retinal glial cells marker), also Wip1 and NF-κB.

**Results:**

ERMs were obtained from 17 eyes of 17 patients with PDR (the PDR group) and 9 eyes of 9 nondiabetic patients (the control group) with iERMs. Our results showed high expression levels of Wip1 mRNAs in ERMs after PDR, but low in iERMs. In addition, both immunohistochemistry and immunofluorescence assay showed strong immunoreactivity for Wip1 in PDR ERMs. Furthermore, Wip1 and GFAP were coexpressed in PDR membranes. Finally, the expression of Wip1 was paralleled with NF-κB.

**Conclusion:**

These data support the notion that Wip1 contributes to the formation of the ERMs in PDR membranes via NF-κB signaling.

## INTRODUCTION

Diabetic retinopathy (DR) is one of the leading causes of decreased vision and blindness in the working-age population of developed countries [1, 2]. Proliferative diabetic retinopathy (PDR) is the more advanced form of DR, characterized by outgrowth of epiretinal membranes at the vitreoretinal interface [3]. These epiretinal membranes (ERMs) may cause distortion of the retinal architecture, vascular leakage, secondary retinal edema and rhegmatogenous retinal detachment [4].

Glial cell proliferation following PDR is suggested to play a crucial role in ERMs formation [4–6]. Glial cell proliferation involves a complex cross-talk among retinal glial cells, including Müller cells and astrocytes [5, 6]. Commonly, the signs of glial cell proliferation are increased immunoreactivity for glial fibrillary acidic protein (GFAP) and glial cell hypertrophy, as well as proliferation and migration of the retinal glial cells [7]. Meanwhile, there is increasing evidence that chronic, low-grade subclinical inflammation play a considerable role in the pathogenesis of DR [8–10]. Furthermore, numerous studies have demonstrated various trophic factor receptors and transcription factors, such as nuclear factor kappa B (NF-kB), are involved in ERMs formation [6, 11, 12]. Unfortunately, the exact mechanisms that regulate the formation of epiretinal membranes in PDR are still incompletely understood.

Wild-type p53-induced phosphatase 1 (Wip1), which is encoded by the PPM1D gene, plays a key role in stress signaling [13]. Wip1 dephosphorylates multiple target proteins in response to various stresses, tumorigenesis and aging [14]. Recent work has demonstrated that Wip1 promotes cell cycle progression, cell survival and proliferation [15]. Importantly, Wip1 might be involved in glia proliferation and inflammation [16, 17]. Additionally, Lowe et al. [18] found that NF-κB p65 was a positive regulator of Wip1 expression. However, little is known about the expression of Wip1 in ERMs of PDR.

The aims of the present study were to investigate the expression and distribution of Wip1 in diabetic patients with PDR with ERMs. Moreover, the colocalization of Wip1 and NF-κB p65 was analyzed by double-staining immunohistochemistry of ERMs.

## RESULTS

### Patient information

Table 1 includes the clinical and demographic Characteristics of patients enrolled in the study. ERMs were obtained from 17 eyes of 17 patients with PDR (the PDR group) and 9 eyes of 9 nondiabetic patients (the control group) with idiopathic epiretinal membranes (iERMs). The patients in the PDR group were 8 females and 9 males whose ages ranged from 37 to 71 years, with a mean of 57.32 ± 1.21 years. The duration of diabetes ranged from 2 to 35 years, with a mean of 12.11 ± 5.39 years. The patients in the control group were 4 females and 5 males (*P* = 1.0) whose ages ranged from 33 to 71 years, with a mean of 58.01 ± 3.61 years (*P* = 0.47). Nine patients in the PDR group and three patients in the control group had hypertension (*P* > 0.42). The fasting blood glucose in the control group ranged from 4.6 to 8.9 mM, with a mean of 5.98 ± 0.25 mM (*P* < 0.01). The diagnoses of the samples in the PDR group included vitreous hemorrhage (*n* = 11), traction retinal detachment (*n* = 13), and panretinal photocoagulation history (*n* = 9). In the control group, no patient had vitreous hemorrhage, traction retinal detachment, and panretinal photocoagulation history. The patients in both groups were enrolled consecutively from March 2014 to December 2015.

**Table 1:** Baseline patient characteristics from epiretinal membranes samples

	PDR group (*n*=17)	Control group (*n*=9)	*p* value
Age (years)	57.32 ± 1.21	58.01 ± 3.61	0.47*
Female Gerder, n (%)	8	4	1.00#
Duration of diabetes (years)	12.11 ± 5.39	N.A.	N.A.
hypertension	9	3	0.42#
Fasting blood glucose, mmol/L	10.09 ± 0.64	5.98 ± 0.25	<0.01*
Vitreoretinal condition:			
Vitreous hemorrhage	11/6	0/9	
Traction retinal detachment	13/4	0/9	
Panretinal photocoagulation history	9/8	0/9	

Data are expressed as the mean ± standard deviation or the median and range. Indicates statistically significant result compared to the corresponding data in the control group as shown in Table 1 (*P<0.05*). * Independent sample t-test. ^#^ χ^2^ test. N.A. = Not applicable.

### mRNA level of Wip1 was higher in the PDR group

**Figure 1:**
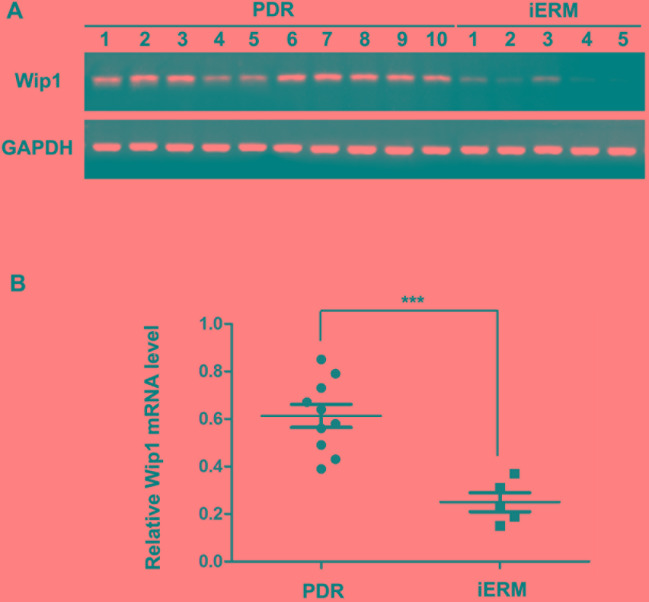
qRT-PCR analysis of Wip1 expression in ERMs derived from patients **A**. cDNA derived from ERMs with PDR and iERMs were analyzed by PCR using specific primers for Wip1. As controls for cDNA integrity, the cDNA was also amplified with specific primers for GAPDH. **B**. Relative mRNA level represented a ratio between the amount of target gene and the amount of GAPDH control. The data are mean ± SEM (***p < 0.01).

Wip1 mRNAs were detected in all ERMs obtained from the PDR group and the control group by qRT-PCR analysis. Glyceraldehyde-3-phosphate dehydrogenase (GAPDH) was amplified as an loading control to compare the relative abundance of PCR products. As shown in Figure 1, in the ERMs, the mRNA level of Wip1 was significantly higher in the patients with diabetes compared to the nondiabetic group. The mean relative mRNA level of Wip1 in PDR groups is 0.62 (range from 0.38 to 0.86), while it is 0.25 in iERM groups (range from 0.14 to 0.36). There is a statistically significant difference in Wip1 mRNA expression between PDR and iERM patients, *P* < 0.01.

### Protein expression of Wip1 was higher in the PDR group

**Figure 2:**
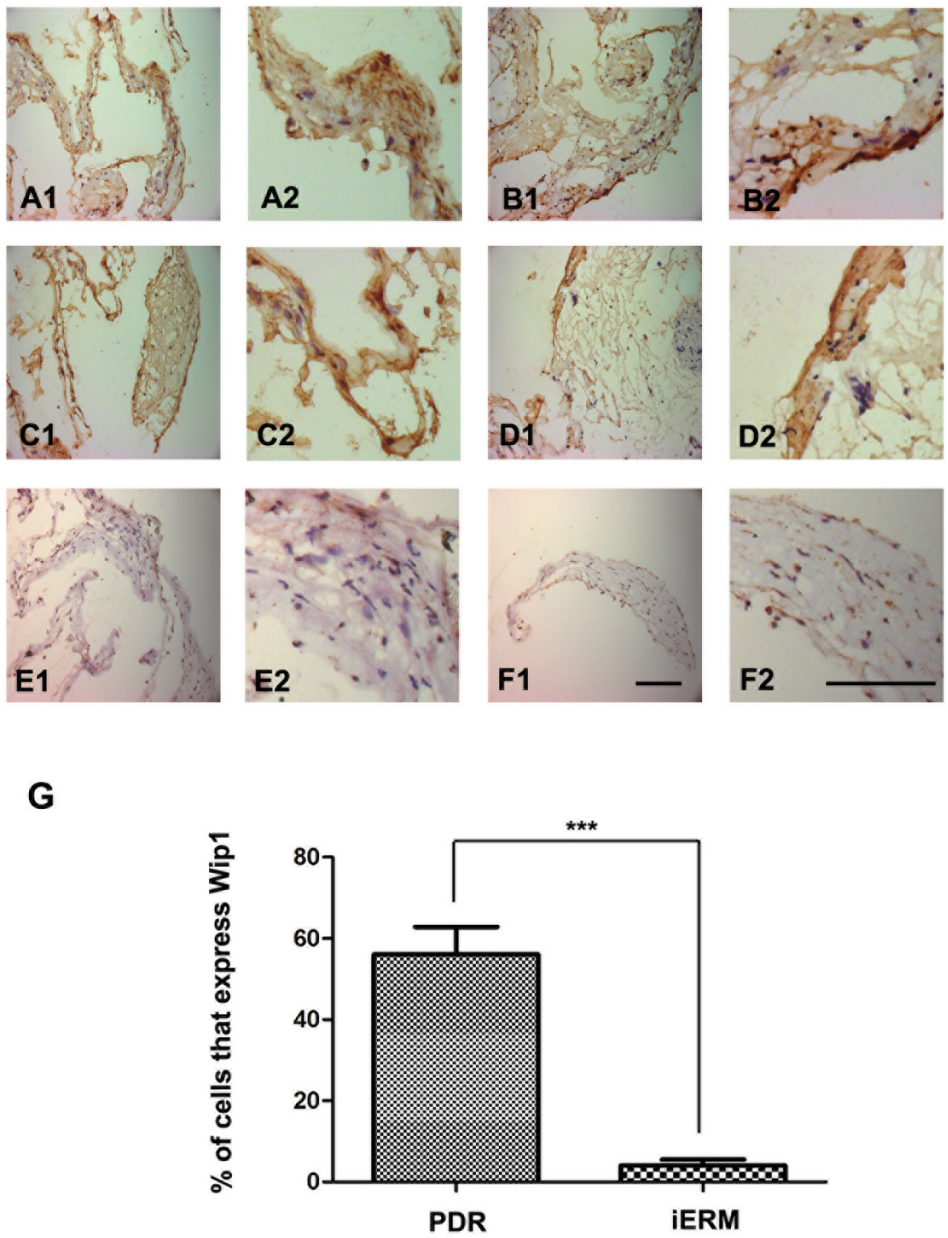
Immunohistochemical epressions of Wip1 in ERM with PDR and iERM samples *Low* (40×) and *higher* (100×) *power views of* Wip1 in iERM samples in PDR group **A**.-**D**. and control group **E**.-**F**. The number of positive cells was scored, and the percentages of Wip1 positive cells were used for statistical comparison **G**. The data were means ± SEM (*n* = 3; ***p < 0.01, significantly different from the control group).

Immunohistochemical analysis was performed to identify the Wip1 protein expressions in both groups. We confirmed weak expression of Wip1 in control group (Figure 2 E1-F2), with a mean number of 3.8 ± 2.5 (Figure 2G). In the PDR group, Wip1 expressions were detected in all frozen sections (Figure 2 A1-D2), with a mean number of 57.2 ± 6.9 (Figure 2G).

**Figure 3:**
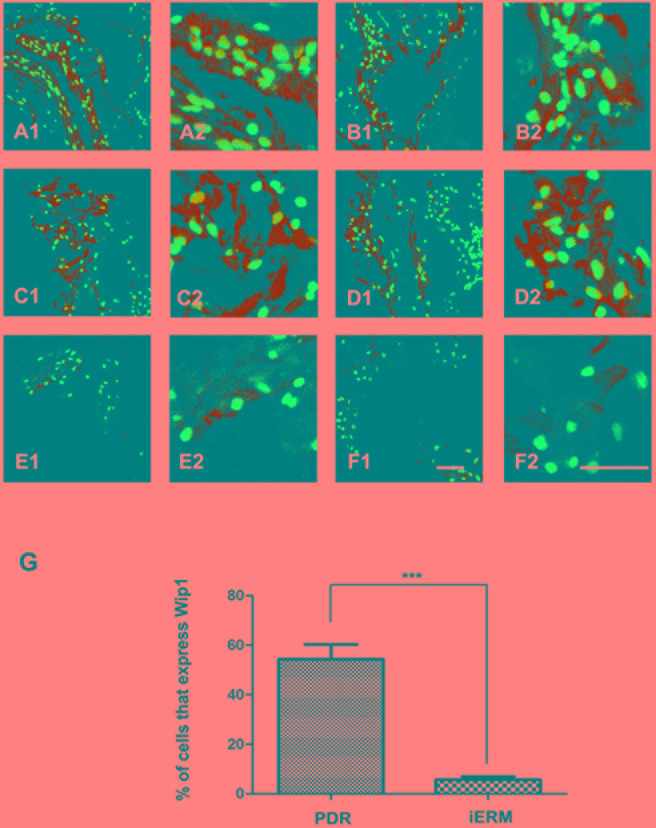
Immunofluorescence expressions of Wip1 in ERM samples *Low* (40×) and *higher* (100×) *power views of* Wip1 in iERM samples in PDR group **A**.-**D**. and control group **E**.-**F**. The number of positive cells was scored, and the percentages of Wip1 positive cells were used for statistical comparison **G**. The data were means ± SEM (*n* = 3; ***p < 0.01, significantly different from the control group).

Consistent with the results of immunohistochemistry, strong immunoreactivity for Wip1 was detected in the PRD group in all membranes by immunofluorescence assay (Figure 3 A1-D2), with a mean number of 55.7 ± 8.2 (Figure 3G). Unlike the PDR group, there was no or weak immunoreactivity for Wip1 in control group (Figure 3 E1-F2), with a mean number of 4.2 ± 1.3 (Figure 3G).

### Co-location of Wip1 and GFAP was detected in PDR group

**Figure 4:**
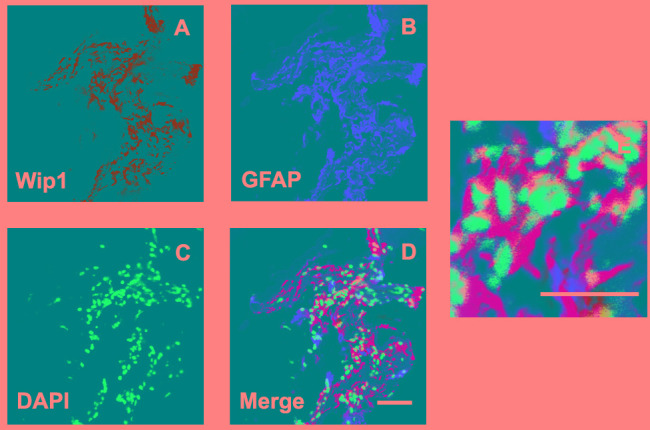
Double labeling immunofluorescent for Wip1 and GFAP in ERM Coexpression of Wip1 **A**. and GFAP **B**. in the ERM derived from a PDR patient. **C**. Nuclei were stained using DAPI. **D**. Merge, magnification 40×. **E**. Merge, magnification 100×. GFAP, glial fibrillary acidic protein(glial cell marker); DAPI, 4′,6-diamidino-2-phenylindole.

**Figure 5:**
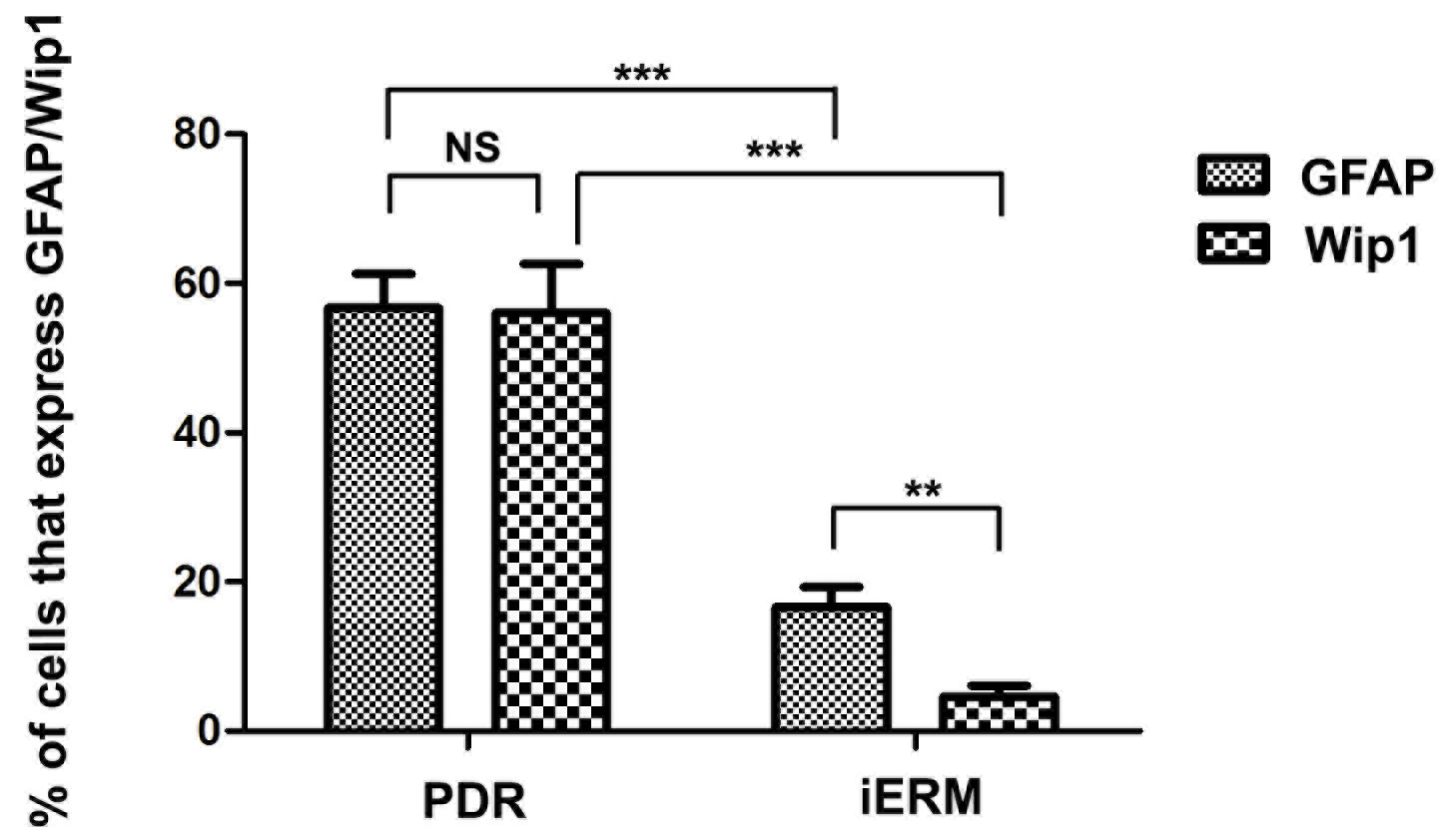
The number of positive cells for GFAP and Wip1 was scored in both groups, and the percentages of positive cells were used for statistical comparison GFAP, glial fibrillary acidic protein. ***p < 0.01, **p < 0.05; NS, non-significant.

Since retinal glial cells are one of the major cellular components of ERMs in PDR, we further detected the co-location of Wip1 and GFAP (retinal glial cells marker). In serial sections, the distribution of glial cells expressing GFAP was similar to the distribution of cells expressing Wip1 in the PRD group in all membranes. Double immunofluorescent staining confirmed that the great majority of these Wip1 cells were GFAP-positive glial cells (Figure 4). The mean numbers of ERMs expressing Wip1 and GFAP (55.8 ± 9.6 and 57.8 ± 7.4, respectively) in PDR group were significantly higher in iERMs (6.7 ± 1.5 and 17.1 ± 3.2, respectively) (*P* < 0.05). Significant correlations were detected between the numbers of Wip1- and GFAP-positive cells in the ERMs after PDR (r = 0.81, *P* < 0.01) (Figure 5).

### Co-location of Wip1 and NF-κB was detected in PDR group

**Figure 6:**
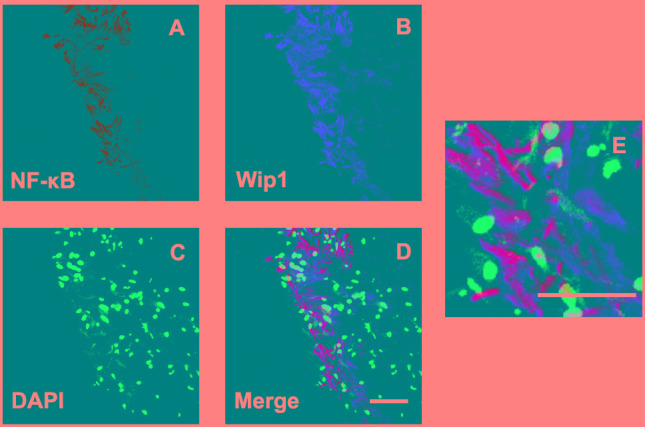
Double labeling immunofluorescent for NF-kB and Wip1 in ERM Coexpression of NF-kB **A**. and Wip1 **B**. in the ERM derived from a PDR patient. **C**. Nuclei were stained using DAPI. **D**. Merge, magnification 40×. **E**. Merge, magnification 100×. NF-kB, nuclear factor kappa B; DAPI, 4′,6-diamidino-2-phenylindole.

Previous studies had demonstrated that NF-κB was involved in the formation of glial cell components of ERMs in PDR. Meanwhile, it has found that NF-κB is a positive regulator of Wip1. Therefore, we further detected the co-location of Wip1 and NF-κB. According to the results of immunofluorescence assay, most of Wip1 positive cells were double labeled with NF-κB (Figure 6). The mean numbers of ERMs expressing Wip1 and NF-κB (57.9 ± 6.8 and 56.7 ± 7.8, respectively) in PDR group were significantly higher in iERMs (4.9 ± 1.2 and 5.3 ± 2.3, respectively) (*P* < 0.05). Significant correlations were detected between the numbers of Wip1- and NF-κB-positive cells in the ERMs after PDR (r = 0.78, *P* < 0.01) (Figure 5).

**Figure 7:**
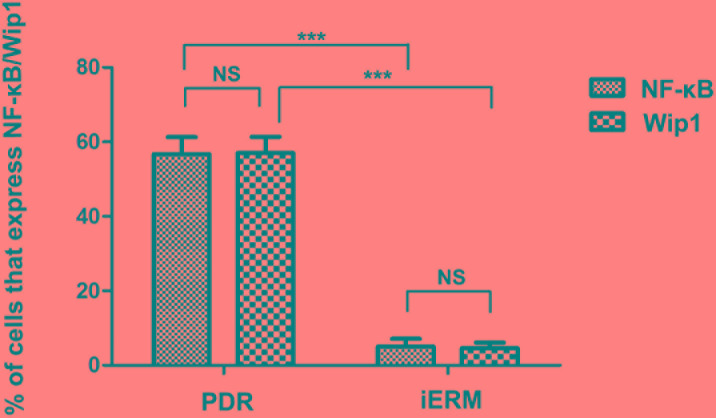
The number of positive cells for NF-kB and Wip1 was scored in both groups, and the percentages of positive cells were used for statistical comparison NF-kB, nuclear factor kappa B. ***p < 0.01; NS, non-significant.

## DISCUSSION

In the present study, we have revealed the expression profiles and location of Wip1 in ERMs. Our results show high expression levels of Wip1 mRNAs in ERMs after PDR, but low in iERMs. In addition, both immunohistochemistry and immunofluorescence assay showed strong immunoreactivity for Wip1 in PDR membranes. Furthermore, Wip1 and GFAP (retinal glial cells marker) were coexpressed in PDR membranes, suggesting Wip1 mainly located in retinal glial cells. Finally, the expression of Wip1 was paralleled with NF-κB. These data support the notion that Wip1 contributes to the formation of the ERMs in PDR membranes *via* NF-κB signaling.

Although the pathogenesis of DR is still not fully understood, DR may have components of chronic inflammation [8, 10]. Epidemiologic evidence indicates that the occurrence of DR and its complications is closely related to the appearance of inflammatory biomarkers [19]. DR has increased serum levels of inflammatory markers (e.g. C-reactive protein, interleukin-6 (IL-6), and tumor necrosis factor-alpha) [20]. Numbers of studies have confirmed that chronic inflammation can stimulate the proliferation of glial cells, which is the one of the major components of ERMs in PDR [21, 22]. Therefore, the proliferation of glial cells and inflammation might be the important points of penetration to explore the pathogenesis of ERM after PDR.

NF-κB has long been considered a prototypical proinflammatory factor present in many cell types that mainly regulates proinflammatory cytokine production, leukocyte recruitment, or cell survival, which are essential for the inflammatory response [23]. The activation of retinal glial cells in the onset of various inflammatory retinal diseases has been linked to the activation of the NF-κB signaling pathway [24]. Activated glial cells can induce the up-regulation of numerous NF-κB target genes including pro-inflammatory cytokines (e.g. TNF-α, IL-1βand IL-6), which are important contributors to the pathological process of DR and the formation of ERMs [6]. There is compelling clinical, histological and experimental evidence that any type of intraocular inflammation can cause ERMs [25]. Meanwhile, it has been confirmed that NF-κB can contribute to the proliferation of glial cells [26]. Importantly, previous study has confirmed that NF-κB mRNA expression levels in ERMs from patients with PDR was significantly higher than those in iERMs [12]. Consistent with previous study, we found strong immunoreactivity for NF-κB in ERMs in the PRD group. These results suggest that NF-κB may be involved in the formation of ERMs after PDR.

Wip1 is one of the members of the Ser/Thr PP2C family, which is encoded by PPM1D gene on chromosome 17q23-24. Lowe et al. [18] have found that PPM1D is a transcriptional target of NF-κB in breast cancer cells. Activation of NF-κB can significantly up-regulate Wip1 protein expression at both the mRNA and protein levels by directly binding to the PPM1D promoter region in LPS and TNF-a-treated splenic B cells [27]. Meanwhile, Wip1 was shown to negatively regulate the expression of NF-κB [28]. These studies described a negative feedback loop involving Wip1 and NF-κB. In the present study, we found that transcriptional and protein expression levels of Wip1 was significantly increased and co-location with NF-κB in ERMs from patients with PDR. Furthermore, the results of analysis of the phenotype of Wip1^−/−^ mice show that Wip1 may involve in the regulation of inflammation [28]. Wip1^−/−^ mice are more susceptible to infection due to the presence of abnormal lymphoid structure and defective T- and B- cell responses [28]. Meanwhile, Wip1 played an important role in regulation of cell proliferation [29, 30]. Overexpression of Wip1 is observed in human gliomas, and PPM1D silencing suppresses proliferation of human glioma cells. In this study, we observed that Wip1 was mainly located in GFAP-positive (retinal glial cells marker) cells, suggesting Wip1 may also be involved in the glial cells proliferation in ERMs in the PRD group.

In conclusion, these data could support the hypothesis that the interactions of NF-κB and Wip1 are involved in the formation of ERMs in PDR. Additional studies are needed to clarify the in-depth mechanisms by which NF-κB/Wip1 signaling pathways regulate inflammatory response and glial cell proliferation in ERMs after PDR, particularly with respect to cross-talk among cellular signaling pathways.

## MATERIALS AND METHODS

### Subjects and sample collection

All ERMs were obtained following approval by the Ethics Committee at People's Hospital of Guangxi Zhuang Autonomous Region, and in accordance with the guidelines of the Declaration of Helsinki for research involving human tissue. Informed consent was obtained from all patients. ERMs samples were collected from patients with PDR (PDR group) or non-diabetic patients with iERMs (control group) during pars plana vitrectomy for the repair of traction retinal detachment or combined traction/rhegmatogenous retinal detachment. The membranes peeled and removed from the retina were fixed in a test tube containing 4% paraformaldehyde (PFA), and were subsequently embedded in optimum cutting temperature (OCT) compound for immunohistochemistry and immunofluorescence.

### Real-time PCR analysis

Total RNA was extracted and purified from frozen specimens using the Trizol reagent (Invitrogen Corporation, Carlsbad, CA, USA) and then reverse transcribed to synthesise complementary DNA (cDNA) according to the manufacturer's protocol. The housekeeping gene GAPDH was used as an internal loading control. The sequences of gene specific primers for Wip1 (forward, 5′-GAAGGATGACTTTGTCAG-3′; reverse, 5′-CCCAGACTTGTTCATT AC-3′) and GAPDH (forward, 5′-ACCACAGTCCAT GCCATCAC-3′; reverse, 5′-TCCACCACCCTGTTGCTGTA-3′) were designed using NCBI Primer-BLAST. All specimens were run in triplicate. The relative differences in expression between groups were expressed using optical density normalized with GAPDH, and the relative differences between PDR and iERM groups were calculated and expressed as relative increases.

### Immunohistochemical analysis

Immunoistochemical assay was performed in accordance with previously studys [24, 31]. Briefly, the cryosections (7-μm thick) were cut by a cryostat, mounted on 3-aminopropyltriethoxysilane-coated glass slides, and air-dried at room temperature. Then the cross-sections were fixed in ice-cold acetone and washed with phosphate buffered saline (PBS). The sections were incubated with normal donkey serum for 30 min to block non-specific staining, and then incubated overnight at 4°C with mouse monoclonal anti-Wip1 antibody (1:150; Santa Cruz Biotechnology, Santa Cruz, CA, US).

### Immunofluorescent analysis

The cryosections were first blocked with 10% normal goat serum, 3%(w/v) bovine serum albumin (BSA) and 0.05% Tween-20 in PBS for 2h at RT. Then, the sections were incubated overnight at 4°C in a humidified box with following primary antibodies: mouse monoclonal anti-Wip1 (1:150; Santa Cruz Biotechnology, Santa Cruz, CA, US), rabbit polyclonal anti-NF-kB p65 (1:200; Santa Cruz Biotechnology, Santa Cruz, CA, US), and rabbit polyclonal anti-GFAP (1:200; Sigma Aldrich, St Louis, MO, US). Sections were then incubated with secondary antibodies for 2h at 4°C. The stained sections were examined at ×40 magnifications on a fluorescence microscope (Leica, DM 5000B; Germany).

### Statistical analysis

Immunoreactive cells were counted in five random fields, using an *eyepiece calibrated grid* with 40 magnification. The cells present in an area of 0.33×0.22 mm were counted. Data were expressed as mean values ± standard deviation (SD) and analyzed by the Mann-Whitney test. Pearson correlation coefficients were computed to investigate the linear relationship between the variables investigated. All collected data were analyzed by SPSS software (SPSS, version 13.0, SPSS, Chicago). All ERM samples were tested in triplicate, and statistical significance was accepted at *P* < 0.05.
